# Supplementary material for: Tumor marker elevations in chronic kidney disease: a retrospective study
Source: PeerJ. 2025 Apr 3;13:e19240. doi: 10.7717/peerj.19240 (PMC11972561; doi:10.7717/peerj.19240)
Supplement: Supplemental Information 2 [file peerj-13-19240-s002.docx]

The raw data's Excel workbook contains two worksheets: one for the control group and the other for CKD patients. In this data, **Group 1** refers to CKD patients, while **Group 2** refers to the control group.
